# Supplementary material for: Conditional chemoconnectomics (cCCTomics) as a strategy for efficient and conditional targeting of chemical transmission
Source: eLife. 2024 Apr 30;12:RP91927. doi: 10.7554/eLife.91927 (PMC11060718; doi:10.7554/eLife.91927)
Supplement: Figure 3—source data 3. [file elife-91927-fig3-data3.zip › Figure 3 source data3 part 1.zip!/Figure 3-Source data 3-part1/1063-13-CRISPResso_Report_8cz_fsdr.zip!/CRISPResso_on_1063_14/1a.Read_barplot.pdf]

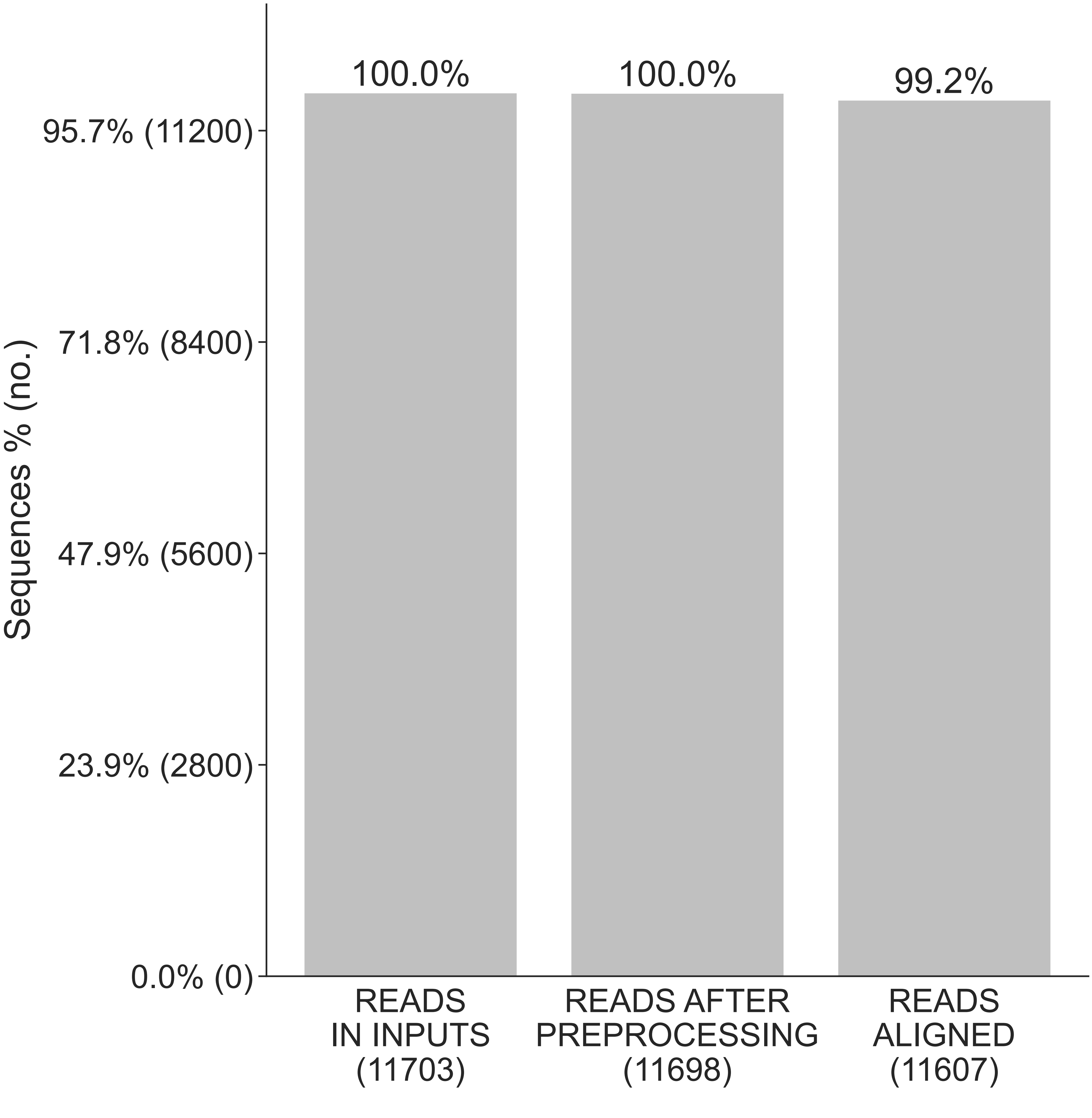

100.0%
100.0%
99.2%
95.7% (11200)
71.8% (8400)
47.9% (5600)
23.9% (2800)
0.0% (0)
Sequences % (no.)
READS IN INPUTS (11703)
READS AFTER PREPROCESSING (11698)
READS ALIGNED (11607)
